# Case Report: Multilevel Ossification of the Ligamentum Flavum in a Patient With Spinal Osteoblastoma

**DOI:** 10.3389/fsurg.2022.890965

**Published:** 2022-06-29

**Authors:** Canada T. Montgomery, Stephen P. Miranda, Ernest Nelson, Katie Louka, MacLean Nasrallah, Paul J. Zhang, Joel Stein, Dmitriy Petrov

**Affiliations:** ^1^ University of Pennsylvania Perelman School of Medicine, Philadelphia, PA, United States; ^2^ Department of Neurosurgery, Hospital of the University of Pennsylvania, Philadelphia, PA, United States; ^3^ Department of Pathology and Laboratory Medicine, Hospital of the University of Pennsylvania, Philadelphia, PA, United States; ^4^ Department of Pathology, Stony Brook University Hospital, Stony Brook, New York, United States; ^5^ Department of Radiology, Hospital of the University of Pennsylvania, Philadelphia, PA, United States

**Keywords:** spinal osteoblastoma, ossification of the ligamentum flavum, myelopathy, neurosurgery, neurological compromise

## Abstract

**Introduction:**

Spinal osteoblastomas are primary benign bone tumors most commonly presenting as diffuse back pain in young adults. Rarely, spinal osteoblastoma is associated with ossification of the ligamentum flavum (OLF), a form of ectopic bone formation, which can present with myelopathy. This report highlights a unique case of a patient with spinal osteoblastoma, associated OLF, and thoracic myelopathy.

**Case Description:**

The patient presented with subtle myelopathy consisting of mid-thoracic back pain, paresthesias, and gait instability. Imaging findings were suggestive of spinal osteoblastoma with multifocal OLF. The patient was consented for thoracic decompression and stabilization at the T6-10 levels. Histopathology confirmed osteoblastoma with associated OLF. At follow up, the patient’s neurological symptoms had completely resolved.

**Conclusion:**

This case describes management for a rare presentation of osteoblastoma with associated OLF and myelopathy. Surgeons should be wary of disproportionate neurological compromise when spinal osteoblastoma is associated with OLF. Further study is required to elucidate the pathogenesis of this condition.

## Introduction

Osteoblastomas are primary benign bone tumors (1–4). They represent approximately 1% of all bone tumors with about 40% being localized to the spine, most commonly the cervical and lumbar regions (1, 2). These tumors are often categorized into two groups, conventional osteoblastomas and aggressive osteoblastomas, based on presentation, imaging, and histology (2). Spinal osteoblastomas most commonly present in young adult males (80% by age 30), usually with dull, diffuse back pain (1–4). Bone scintigraphy is the most sensitive radiographic modality for spinal osteoblastomas, however, plain radiographs, computed tomography (CT) and magnetic resonance imaging (MRI) are often used for initial characterization of the lesion (1). In general, spinal osteoblastomas can display osteoblastic and osteolytic characteristics and usually have a nidus greater than 2 cm (2). Definitive treatment consists of complete resection, and radiation and chemotherapy are considered when complete surgical resection is not possible (1–4).

Ectopic bone formation, particularly ossification of the ligamentum flavum (OLF), is a rare finding in osteoblastoma (5–7). While other reports of OLF and osteoblastoma present solely with back pain, OLF itself can present with myelopathy and spinal stenosis (5, 7). The etiology of OLF associated with spinal osteoblastoma is largely unknown. Some proposed mechanisms postulate that growth hormones and inflammatory cytokines act as mediators in OLF pathogenesis (8). Here, we present a case of spinal osteoblastoma in which associated OLF and thoracic myelopathy created important implications for surgical management that clinicians should be aware of.

## Case Description

**Figure 1:**
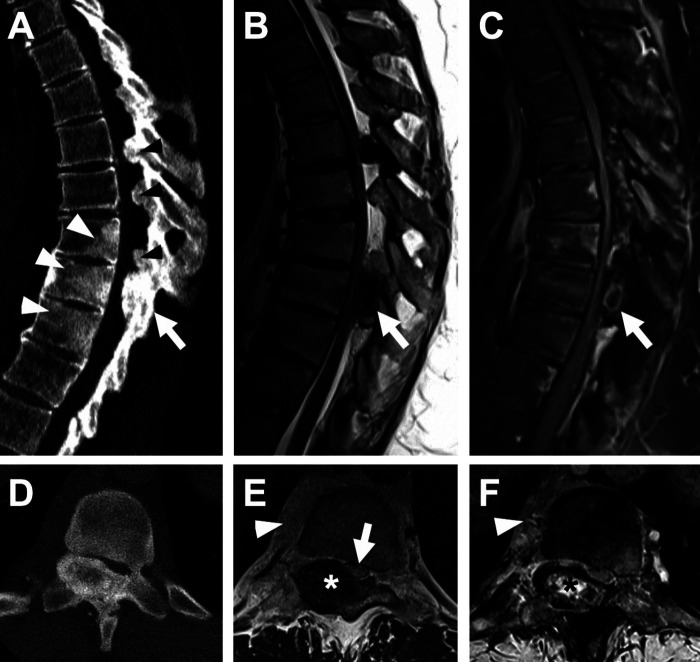
Osteoblastoma with multilevel ligamentum flavum ossification. (**A**) Sagittal CT and (**B**) T2-weighted and (**C**) fat-saturation post-contrast T1-weighted MR images show an expansile osseous lesion (arrow) centered at the right T9 lamina and pedicle with a central ring of enhancement. Note associated regional sclerosis in the T8-10 vertebral bodies (white arrowheads in **A**) and prominent multilevel ligamentum flavum ossification (black arrowheads in **A**). (**D–F**) Corresponding axial images show central lucency within the osseous lesion with heterogenous low T2 signal and enhancement (asterisk), as well as marked canal stenosis and cord compression (arrow in **E**). Note also enhancing right paravertebral soft tissue (arrowhead in **E** and **F**). Ligamentum flavum ossification and epidural lipomatosis also cause significant stenosis at T6-7, T7-8 and T8-9.

A 30-year-old obese male with a history of hypertension, hyperlipidemia and mild scoliosis presented with subtle clinical features of myelopathy. He endorsed one week of left plantar foot paresthesias exacerbated by activity, gait instability and mid-thoracic back pain. On physical examination, he had poor proprioception in his left lower extremity. CT revealed osseous lesions at T7-10 extending into surrounding paraspinal tissues causing severe spinal canal stenosis. MRI of the spine demonstrated an expansile sclerotic osseous lesion centered at the right lamina and pedicle of T9 with central lucency, enhancement and narrowing of the spinal canal at T9-10, as well as multifocal OLF most pronounced at T6-7 and to a lesser degree at T7-8 and T8-9 (Figure 1). Together, these findings were suggestive of osteoblastoma with associated OLF and reactive bone and soft tissue inflammation, often described as the osteoblastoma flare phenomenon (9).

**Figure 2:**
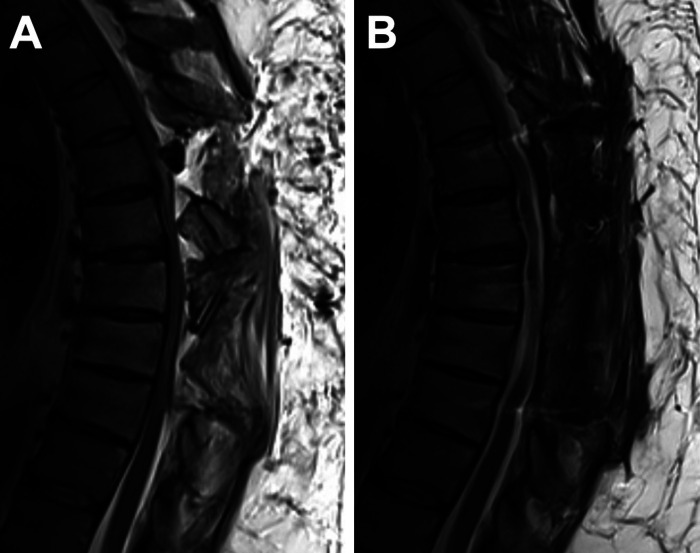
Surgical resection and decompression. (**A**) Sagittal T2-weighted MRI of the thoracic spine after initial resection and decompression, prior to stabilization. (**B**) Three-month follow up sagittal T2-weighted MRI after further decompression and stabilization, without residual spinal stenosis.

The patient consented to surgical intervention and underwent thoracic decompression and stabilization. Wide laminectomies at the T6-10 levels were performed. The ligamentum flavum was found to be significantly calcified, at times requiring high-speed pneumatic drilling for removal. Decompression was carried down through the pedicle at T9 to adequately manage the critical stenosis caused by the bony mass at T9-10. After decompression, T6-10 stabilization was performed. Post-operatively, the patient had mild left lower extremity weakness which improved spontaneously within 24 h (4+/5). At his 3-month follow up visit, the patient had complete resolution of his neurological symptoms with stable imaging findings (Figure 2).

**Figure 3:**
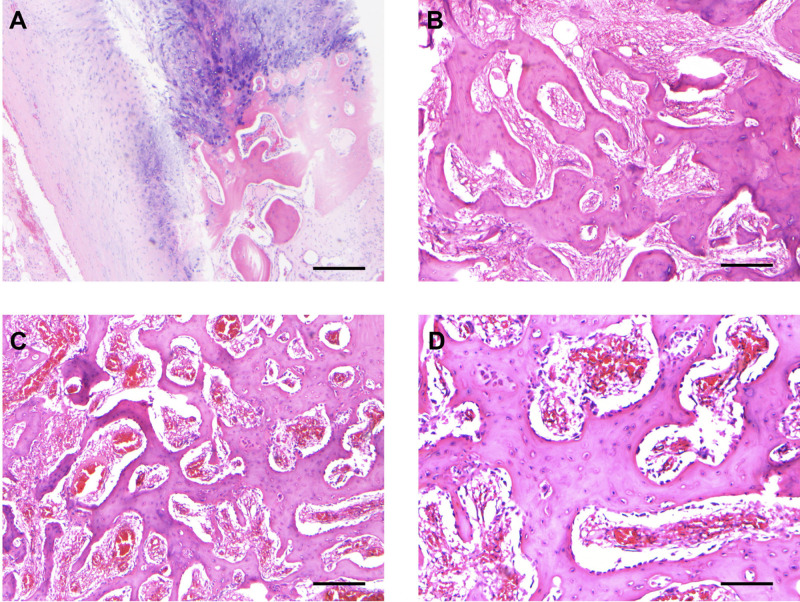
Histopathological characteristics assessed on surgical resection. (**A**) The section shows the reactive, benign chondro-osseous tissue and adjacent fibrous tissue that comprised the ossification of the ligamentum flavum specimen. Scale bar, 250 µm. (**B,C**) Representative sections show the anastomosing trabeculae of woven bone with loose fibrovascular stroma with focal hemorrhage and congested blood vessels, consistent with osteoblastoma. Scale bar, 250 µm. (**D**) Area of tumor with prominent osteoblastic rimming. Scale bar, 125 µm.

The surgical pathology report noted osteoblastoma associated with OLF. The OLF was characterized as reactive, benign chondro-osseous tissue. Histology demonstrated anastomosing trabeculae of osteoid and woven bone with prominent osteoblastic rimming and scattered osteoclastic activity. Loose fibrovascular stroma with focal hemorrhage and vascular congestion was seen between the trabeculae, consistent with the diagnosis of osteoblastoma (Figure 3).

## Discussion

Here, we present a unique case of osteoblastoma with associated OLF and disproportionate spinal cord compromise that may create unexpected implications for surgical management. Clinicians should be wary of thoracic myelopathy associated with osteoblastoma with OLF, even if the patient is ambulatory with grossly full strength on exam, as in this case. The severity of stenosis associated with the bony mass at T9-10 and the degree of OLF calcification seen intraoperatively was more extensive than the patient’s clinical status suggested. Preoperatively, there was low suspicion that the patient would have significant cord compromise given his baseline motor exam and the absence of spinal instability on preoperative testing. Nevertheless, the patient did have mild weakness postoperatively, likely related to the spinal cord manipulation required to achieve adequate decompression at the levels with significant OLF in the setting of existing myelopathy.

Little is known about the presentation, risk factors and pathophysiology of spinal osteoblastoma associated with OLF (5, 6). Again, the rarity of this condition is further highlighted in Table 1 which provides an overview of the few reported cases in the literature. OLF alone has a unique set of risk factors and proposed mechanisms that can provide insight into the development of OLF in the presence of osteoblastoma. For instance, studies have shown a genetic predisposition among East Asian patient populations (8, 10). Additional risk factors for OLF include metabolic disturbances seen in obesity and diabetes mellitus, both of which were present in this patient (8, 11, 12). A proposed explanation is the dysregulation and imbalance of growth factors and inflammatory cytokines, such as bone morphogenetic protein (BMP) and transforming growth factor beta (TGF-beta), which play a role in the development and growth of new cartilage and bone tissue (12). Studies have shown that degenerative intervertebral disks secrete BMP and other inflammatory cytokines that may influence the local microenvironment of nearby structures like the ligamentum flavum (LF) (13, 14). In addition, OLF cells at baseline have a higher expression of osteopontin, beta-catenin, Sox9 and Runx2, which are transcription factors for osteogenesis (13, 14). Therefore, the elevated levels of inflammatory cytokines and growth factors in the local milieu of the LF could induce and maintain osteogenic signaling pathways leading to hypertrophy and OLF (13, 14). BMP-2/4 is of particular interest in the association of osteoblastoma and OLF (15). There have been reports that show increased BMP-2/4 levels are present in osteoblastomas (6). Thus, the mechanism of OLF in osteoblastoma probably involves secretion of BMP-2/4 or other factors stimulating or responding to surrounding inflammation. Patients predisposed to OLF either by genetics, biomechanics, obesity, or diabetes may then develop prominent OLF as in our illustrative case. Ultimately, further research is required to better understand the mechanism by which ectopic bone formation occurs in this disease.

**Table 1:** Literature review of osteoblastoma & osteoid osteoma cases with associated ossification of the ligamentum flavum.

Article	Patient characteristics	Radiography	Operation	Treatment course
Kojima et al., 1992 (5)	22-year-old male with obesity and difficulty climbing stairs. Spastic paraparesis below T11 on exam.	CT myelogram showed obstruction from T10-11	Surgical excision of ossified ligaments from T8-11	Elevated fasting serum insulin and glucose tolerance test. Tetracycline administered prior to surgery to detect progressive OLF. Histopathology confirmed osteoblastoma. Motor and sensory function deficits gradually resolved after surgery.
	22-year-old male with lower back pain with activity and at night that responded well to NSAIDs. Spinous process tenderness at T10 on exam. Ossification of anterior longitudinal ligament and OLF seen in family members.	Plain radiographs and CT showed enlargement of the right pedicle and superior articular process of T11 with OLF from T10-L1.	Surgical resection of lesion at T11 and OLF from T10-12.	All labs were normal. Tetracycline administered prior to surgery to detect progressive OLF. Histopathology confirmed osteoid osteoma. Pre-operative pain resolved after surgery.
Okuda et al., 2001 (6)	32-year-old male with low back pain worse at night and relieved with NSAIDs. Unremarkable exam.	Plain radiographs showed scoliosis and sclerotic changes of the T9 pedicle. Bone scintigraphy showed uptake in left inferior facet of T9. CT showed an osteolytic tumor with a sclerotic rim and focal OLF. T2-weighted MRI showed hyperintense lesion surrounded by hypointense area and hypointense OLF.	Left hemilaminectomy of T9 with complete excision and resection of the left superior facet of T10.	Routine labs normal. Histopathology confirmed osteoblastoma. Immunohistochemistry demonstrated bone morphogenetic protein (BMP-2/4) expression. Pre-operative pain resolved after surgery.
	25-year-old male with low back pain. Unremarkable exam.	CT showed an osteolytic tumor in the right superior facet of L1 with OLF on both sides of L2. T2-weighted MRI showed intermediate intensity signal of the tumor; OLF not clearly seen.	Right hemilaminectomy of L1 with lesional excision and resection of the right superior facet of L2.	Routine labs normal. Histopathology confirmed osteoblastoma. Pre-operative pain resolved after surgery.
	27-year-old female with low back pain. Unremarkable exam.	Plain radiographs showed cortical expansion of the right inferior facet of L3. CT showed an osteolytic tumor at the right inferior facet of L3 with OLF. T2-weighted MRI showed intermediate intensity signal of tumor with surrounding hyperintensity; OLF not clearly seen.	Right hemilaminectomy of L3 with lesional excision.	Routine labs normal. Histopathology confirmed osteoblastoma. Pre-operative pain resolved after surgery.

In conclusion, surgeons need to be aware of the implications of OLF in the setting of osteoblastoma and plan surgical interventions accordingly. Spinal osteoblastoma associated with OLF is rare with limited information in the literature to help guide management. This case emphasizes the degree of neurological compromise that may be seen in osteoblastoma with OLF and myelopathy, and close attention should be paid during patient positioning and throughout surgery.

## Data Availability

The original contributions presented in the study are included in the article/Supplementary Material, further inquiries can be directed to the corresponding author/s.
